# Stage 1 Registered Report: sex differences in anticoagulation and stroke outcomes among patients with atrial fibrillation: a registry-based cohort study

**DOI:** 10.1093/braincomms/fcag386

**Published:** 2026-10-22

**Authors:** Benedikte L Nygaard, Soffien C Ajmi, Camilla J H Normand, Kathinka D Kurz, Lars Fjetland, Friedrich M W Kurz

**Affiliations:** Department of Neurology and Neuroscience Research Group, Stavanger University Hospital, 4068 Stavanger, Norway; Department of Neurology and Neuroscience Research Group, Stavanger University Hospital, 4068 Stavanger, Norway; Faculty of Health Sciences, University of Stavanger, 4036 Stavanger, Norway; Faculty of Health Sciences, University of Stavanger, 4036 Stavanger, Norway; Division of Internal Medicine, Stavanger University Hospital, 4068 Stavanger, Norway; Stavanger Medical Imaging Laboratory (SMIL), Department of Radiology, Stavanger University Hospital, 4068 Stavanger, Norway; Department of Electrical and Computer Engineering, University of Stavanger, 4036 Stavanger, Norway; Stavanger Medical Imaging Laboratory (SMIL), Department of Radiology, Stavanger University Hospital, 4068 Stavanger, Norway; Department of Clinical Medicine, University of Bergen, 5020 Bergen, Norway; Department of Neurology and Neuroscience Research Group, Stavanger University Hospital, 4068 Stavanger, Norway; Department of Clinical Medicine, University of Bergen, 5020 Bergen, Norway

**Keywords:** atrial fibrillation, stroke, thrombectomy, anticoagulation, sex differences

## Abstract

Women with atrial fibrillation have a higher risk of ischaemic stroke and worse functional outcomes than men but may be less likely to receive adequate anticoagulation. Preliminary single-centre findings in patients treated with endovascular thrombectomy for large vessel occlusion suggest that women may be under-anticoagulated prior to stroke onset. This Stage 1 Registered Report outlines a prespecified analysis plan to test sex-based differences in anticoagulation, stroke characteristics, treatment and outcomes in a prespecified confirmatory population and to explore whether similar patterns are observed at the national level. Using a retrospective cohort design, we will analyse local data from Stavanger University Hospital (2009–22) and national data from the Norwegian Stroke Registry. Confirmatory analyses will include patients with atrial fibrillation and large vessel occlusion treated with endovascular thrombectomy. Sex differences in anticoagulation, stroke severity, treatments and outcomes will be examined using chi-square tests and prespecified regression models; equivalence testing will be applied where appropriate. Exploratory analyses will assess age, comorbidity and healthcare delivery as potential mediators and moderators. No confirmatory analyses have been conducted.

## Teaser

Nygaard *et al*. describe a preregistered plan to examine sex differences in anticoagulation status and outcomes among patients with atrial fibrillation presenting with large vessel occlusion stroke treated with endovascular thrombectomy, using Norwegian hospital and national registry data.

## Graphical Abstract

**Graphical Abstract:**
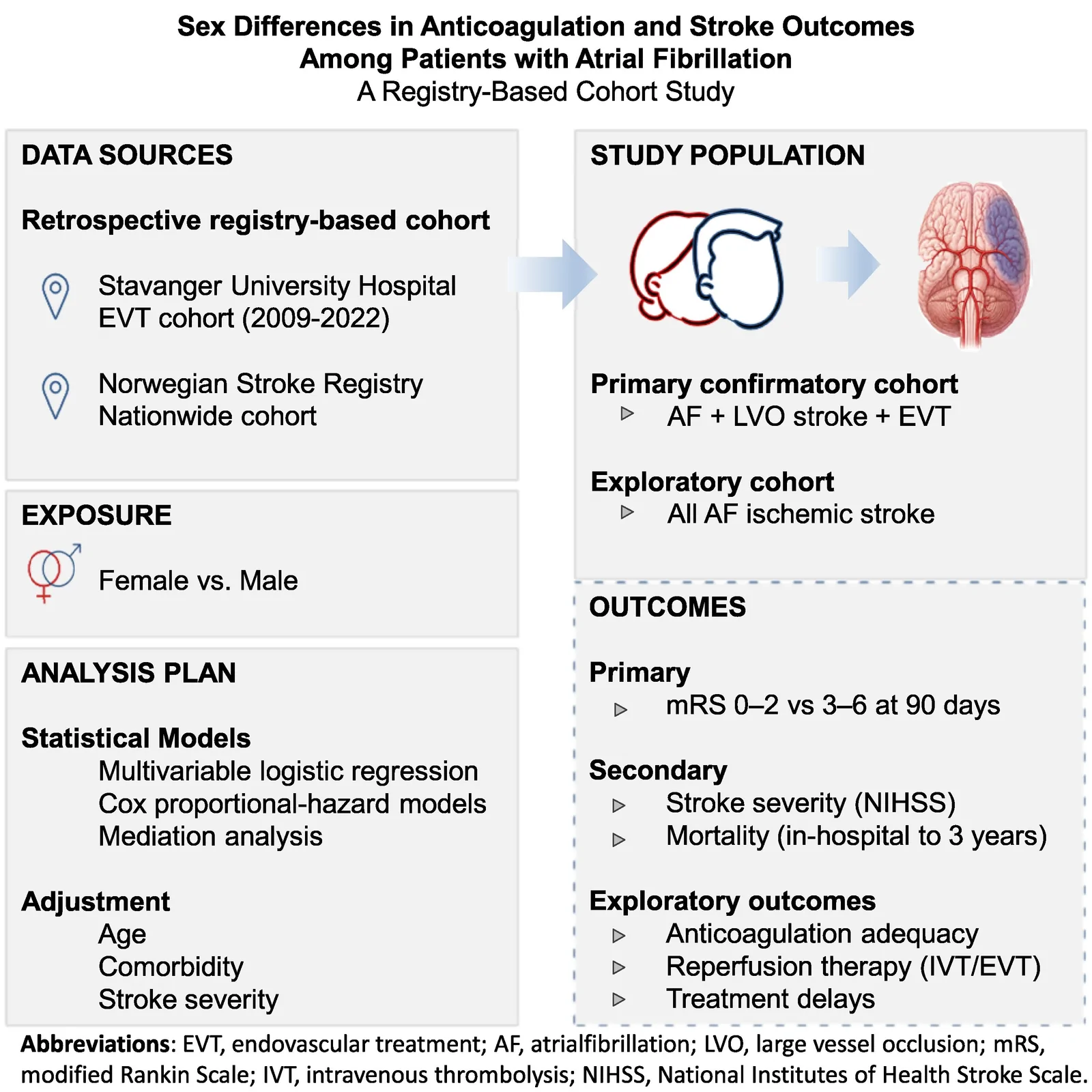

## Stage 1 statement

This submission is a Stage 1 Registered Report. No confirmatory analyses have been conducted beyond feasibility checks.

Rationale for Registered Report: this study uses observational registry data in which cohort definitions, outcome operationalization, covariate adjustment choices, and missing data handling can substantially influence conclusions. Registering hypotheses and the analysis plan before confirmatory analyses begin and prior to access to national registry data reduces analytic flexibility and selective reporting. This is particularly relevant for sex-difference research, where multiple plausible modelling choices can produce divergent inferences. Stage 1 in-principle acceptance therefore strengthens the credibility of the confirmatory findings irrespective of statistical significance.

## Introduction

Atrial fibrillation (AF) is one of the most important modifiable risk factors for ischaemic stroke, particularly among older adults.^1^ Oral anticoagulants (OACs), including vitamin K antagonists (VKAs) and direct oral anticoagulants (DOACs), significantly reduce stroke risk and mortality in patients with AF.^2,3^ However, numerous studies indicate that women are less likely than men to receive adequate anticoagulation despite a higher baseline stroke risk and poorer outcomes after ischaemic events.^4-6^

Large vessel occlusion (LVO) strokes, caused by the blockage of a major cerebral artery, are the most severe stroke subtypes and often require endovascular treatment (EVT). Sex-based disparities in anticoagulation status among patients presenting with LVO strokes may contribute to differences in stroke severity, access to treatment and clinical outcomes. In preliminary observations from Stavanger University Hospital, women with AF were more often under-anticoagulated prior to stroke compared with men, even though EVT treatment rates and outcomes appeared similar.

This study aims to test specific, preregistered hypotheses regarding sex differences in anticoagulation, stroke management and outcomes in patients with AF. We will use a preregistered confirmatory framework, with national registry data used to explore the generalizability of the confirmatory findings.

## Study aims and hypotheses

### Primary aim (confirmatory)

To assess whether sex differences exist in stroke risk factors, clinical presentation, treatment and outcomes among patients with AF, using a preregistered confirmatory design.

Primary analyses will be conducted in patients with AF presenting with LVO stroke treated with EVT, as this represents a clinically homogeneous group in which treatment disparities are highly relevant. To assess generalizability to the broader AF stroke population, exploratory analyses will also include all patients with AF and ischaemic stroke recorded in the Norwegian Stroke Registry.

### Primary hypothesis (H1)

Among adults with AF presenting with large vessel occlusion ischaemic stroke treated with endovascular thrombectomy, women differ from men in prespecified stroke risk factors, clinical presentation and clinical outcomes, including stroke severity at admission, 3-month functional outcome, and mortality.

### Null hypothesis (H0)

There are no sex-based differences in prespecified stroke risk factors, clinical presentation, treatment or outcomes among adults with AF presenting with LVO stroke treated with EVT.

### Secondary aims (exploratory)

- **H2:** Women are older at stroke onset and have a higher burden of cardiovascular comorbidities compared with men.
  Operational definitions for H1 versus H2 variable sets: In H1, ‘stroke risk factors’ refer to a prespecified set of baseline variables representing established risk factors for ischaemic stroke in AF (hypertension, diabetes mellitus, prior stroke/TIA, vascular disease and age strata as captured by available registry variables). In H2, ‘cardiovascular comorbidities’ refer to a broader prespecified set reflecting overall cardiovascular disease burden and, where feasible, a comorbidity summary measure. H2 is exploratory and is not used to redefine or expand the H1 confirmatory variable set.
- **H3:** Women experience longer pre-hospital and in-hospital delays and are less likely to receive reperfusion therapies [intravenous thrombolysis (IVT) or EVT].
- **H4:** Women with AF are less likely to be adequately anticoagulated prior to stroke onset.
- **H5:** Sex differences in outcome are mediated in part by age, comorbidity burden and differences in healthcare delivery (e.g. time to treatment, access to EVT).

## Materials and methods

### Ethics statement

Ethical approval has been granted by the Regional Committee for Medical and Health Research Ethics (REK Vest, reference 217700), the Norwegian Centre for Research Data (NSD) and the data protection officer at Stavanger University Hospital for the retrospective analysis of stroke patients with AF. This approval covers the use of local data from Stavanger University Hospital and linkage with the Norwegian Stroke Registry and the Norwegian Prescription Registry, with a focus on age- and sex-based differences in anticoagulation, treatment and outcomes.

Following Stage 1 in-principle acceptance, a formal amendment will be submitted to REK to expand the ethical approval to include analyses of long-term mortality (1, 2 and 3 years) through linkage with the Norwegian Cause of Death Registry and to clarify the use of a Registered Report methodology. No confirmatory analyses involving these extended data will be conducted before the updated REK approval has been granted.

### Study design

This is a retrospective cohort study with a confirmatory primary analysis and several exploratory secondary analyses. Data will be obtained from two sources:

- **A single-centre EVT cohort** from Stavanger University Hospital (Sept 2009–Dec 2022).^7^
- **The Norwegian Stroke Registry**, encompassing all ischaemic stroke patients nationwide during the same period.

The local dataset includes detailed clinical timelines, imaging results and EVT procedure data. The national dataset provides population-level coverage for exploratory assessment of generalizability and broader patterns.

Open Science Framework Preregistration: The full protocol, hypotheses and statistical analysis plan will be preregistered in the ‘Stiftelsen Dam’ registry on the Open Science Framework before confirmatory analyses begin.

#### Statistical analyses summary

Confirmatory analyses will be performed in adults with AF presenting with LVO stroke treated with EVT. The primary confirmatory endpoint is functional outcome at 3 months (modified Rankin Scale (mRS) 0–2 versus 3–6) modelled using logistic regression with prespecified adjustment for age, baseline stroke severity, comorbidities and acute treatments. Stroke severity at admission (NIHSS) and mortality endpoints will be analysed as secondary confirmatory outcomes using appropriate regression models, including logistic regression at prespecified time points and, where dates are available, Cox proportional hazards models. Missing data will be handled using multiple imputation if key variables exceed 5% missingness, with complete-case sensitivity analyses. Multiplicity across confirmatory outcomes will be controlled using Holm–Bonferroni adjustment (two-sided family-wise alpha 0.05), and effect sizes with 95% confidence intervals will be reported for all endpoints.

An overview of the study timeline, data sources, eligibility criteria, exclusions and final analytic cohorts is shown in Fig. 1.

**Figure 1:**
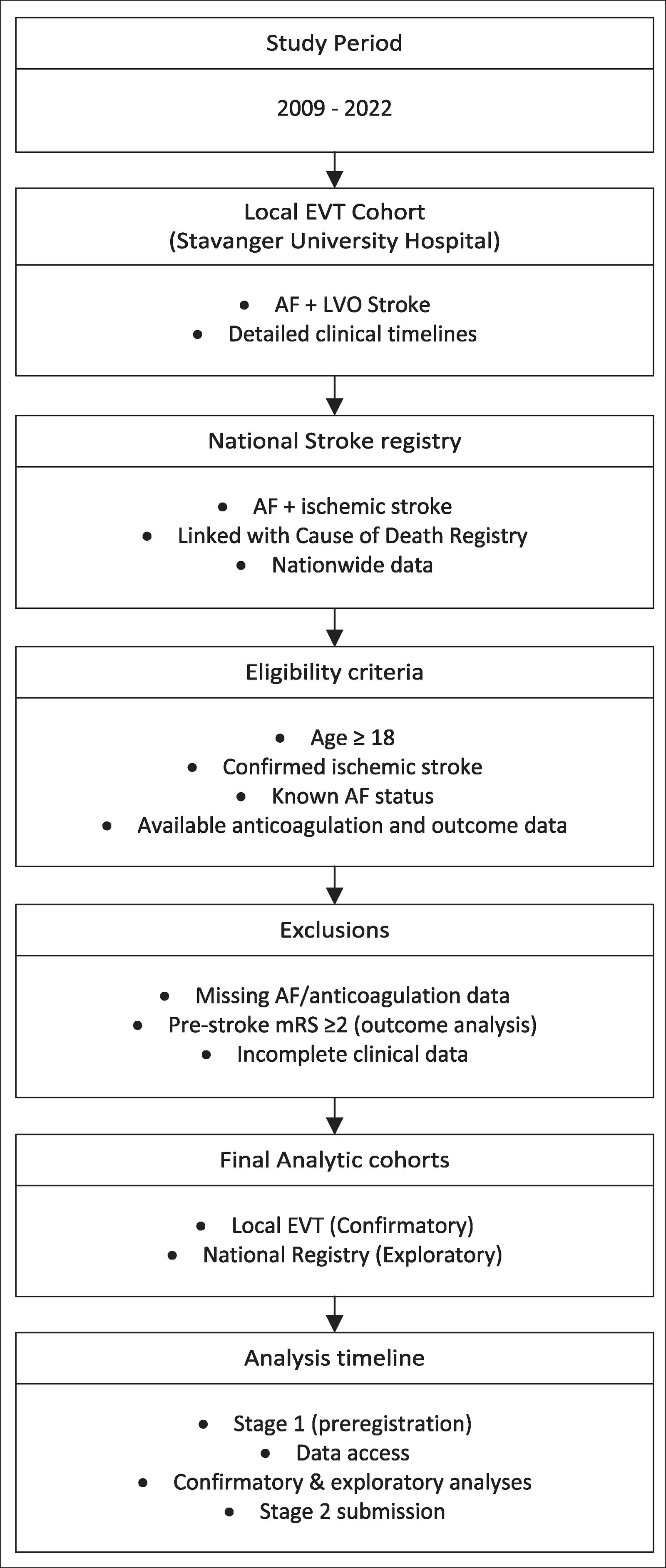
**Study timeline and cohort flow.** Schematic overview of the study population, data sources, eligibility criteria and analytic timeline. Local EVT data from Stavanger University Hospital and national stroke registry data from Norwegian Stroke Registry (2009–22) will be used to examine sex differences in anticoagulation, stroke characteristics, treatment and outcomes. The local EVT cohort will be used for confirmatory analyses, and the national cohort for exploratory assessment and generalizability.

### Participants

#### Inclusion criteria (local and national data)

- Adults (age ≥18 years) with ischaemic stroke recorded in the local or national datasets.
- Confirmed LVO stroke (for local data).
- Treated with EVT (for local data).
- Diagnosis of AF known prior to stroke or diagnosed during hospitalization.

#### Exclusion criteria

- No AF diagnosis.
- Missing data on anticoagulation status.
- Pre-stroke mRS ≥ 2 (for functional outcome analyses).
- Missing 3-month outcome data (for functional outcome analyses).
- Insufficient clinical data for key variables.

Primary analyses will be restricted to patients with AF and LVO stroke treated with EVT. As a planned exploratory extension, we will also examine all patients with AF and ischaemic stroke in the Norwegian Stroke Registry to assess whether observed sex differences generalize beyond the LVO population.

Based on prior Norwegian and international data, LVO accounts for ∼20%–30% of ischaemic strokes, corresponding to an estimated 1500–3000 cases annually in Norway. We expect ∼40–45% female and 55–60% male participants in the study cohort.

### Variables

#### Exposure variable

Patient sex (female or male) is recorded in the hospital and registry datasets. Information on gender identity is not available; analyses will therefore examine sex differences.

Confirmatory outcomes:

- Primary confirmatory endpoint: Functional outcome at 3 months defined as mRS 0–2 versus 3–6.
- Secondary confirmatory endpoints: Stroke severity at admission, assessed using NIHSS.
- Mortality will be assessed at prespecified time points (in-hospital, 3 months, 1 year, 2 years and 3 years) based on hospital records and/or linkage with the Norwegian Cause of Death Registry.

#### Secondary exploratory outcomes

- Adequate anticoagulation at stroke onset
  - Definition of adequate anticoagulation: For vitamin K antagonists, adequate anticoagulation will be defined as a therapeutic international normalized ratio (INR ≥ 2.0) on admission (or the closest available pre-stroke INR). For direct oral anticoagulants, adequate anticoagulation will require documentation of regular prescribed use at a therapeutic dose at the time of stroke and documented last intake within 48 h prior to symptom onset (primary definition). If regular use cannot be established, anticoagulation adequacy will be classified as uncertain/unknown. Sensitivity analyses will evaluate a stricter window (last intake within 24 h) and alternative handling of the uncertain category.
- Treatment received, including IVT and EVT.
- Time to treatment variables: onset-to-door, door-to-imaging, door-to-needle and door-to-groin puncture times.

#### Covariates

- Age, pre-stroke mRS and comorbidities including hypertension, diabetes and previous stroke.
- Health system factors, including weekday versus weekend admission, inter-hospital transfer and year of treatment.

#### Dissemination plan

The Stage 2 Registered Report will present all preregistered confirmatory analyses in a single integrated manuscript, irrespective of the direction or statistical significance of the results. No preregistered confirmatory analyses will be split across multiple publications. Following the completion of Stage 2 reporting, additional analyses using the same datasets may be undertaken to address secondary or exploratory research questions. These manuscripts will be clearly identified as exploratory, will explicitly reference the Registered Report preregistration and will not re-label *post hoc* analyses as confirmatory.

### Statistical analysis plan

#### Primary (confirmatory) analysis

Confirmatory endpoint hierarchy and multiplicity: The primary confirmatory endpoint is a functional outcome at 3 months, defined as binary mRS score 0–2 versus 3–6. NIHSS at admission and mortality endpoints (in-hospital, 3 months, 1 year, 2 years and 3 years) are secondary confirmatory outcomes. To control the family-wise error rate across the confirmatory outcome family, we will apply Holm–Bonferroni adjustment at a two-sided alpha of 0.05 and report both adjusted and unadjusted *P*-values alongside effect sizes and 95% confidence intervals. Baseline sex-based differences in stroke severity, clinical presentation, treatments and outcomes were assessed using chi-square tests for categorical variables and *t*-tests or Mann–Whitney U-tests for continuous variables, as appropriate. Logistic regression will be used to assess the association between sex and favourable functional outcome (mRS 0–2 at 3 months), adjusting for prespecified age, comorbidities, stroke severity and treatment variables.

Mortality analyses: logistic regression models will assess the association between sex and mortality at 3 months, 1 year, 2 years and 3 years. Where exact dates of death are available, time-to-event (survival) analyses will be conducted using Cox proportional hazards models, adjusted for age, stroke severity, comorbidities and treatment variables.

#### Secondary (exploratory) analyses

- Logistic regression will be used to identify predictors of anticoagulation status, stratified by sex.
- Multivariable models including interaction terms (sex × age, sex × comorbidities) will be explored.
- Mediation analyses will be conducted to assess whether age, comorbidity burden and health system factors mediate observed sex differences in outcomes, using bootstrapped indirect effects.
- Subgroup analyses will be performed stratified by age (<75 versus ≥75 years), treatment year and stroke severity.
- Equivalence testing will be considered for selected confirmatory outcomes in the event of null findings, using prespecified equivalence margins.

#### Power calculation

Preliminary data suggest an approximate 10% difference in adequate anticoagulation rates between sexes (70% in men versus 60% in women). With α = 0.05 and 80% power, ∼800 patients with AF would be required to detect this difference. The Norwegian Stroke Registry includes a sufficiently large population to support this level of precision. This calculation is intended to demonstrate feasibility and expected precision rather than to define power for all confirmatory outcomes.

### Deviations from the preregistered analysis plan

Any deviations from this preregistered analysis plan will be transparently documented and justified in the Stage 2 Registered Report. All changes will be clearly distinguished from preregistered confirmatory analyses and reported as exploratory analyses.

### Missing data

Multiple imputations will be conducted if more than 5% of values are missing for prespecified key variables, under a missing-at-random (MAR) assumption. Sensitivity analyses will compare complete-case and imputed results.

### Quality checks

To ensure data validity and the feasibility of the planned analyses, the following checks will be conducted prior to confirmatory analyses:

- Verify that registry data show expected distributions of age and comorbidity by sex at stroke onset.
- Inspect NIHSS and mRS distributions for floor or ceiling effects.
- Cross-validate anticoagulation status against medication records and clinical notes, where available.
- Assess the plausibility and consistency of treatment times and intervention rates.

### Language editing and use of AI tools

Artificial intelligence tools (ChatGPT, OpenAI) will be used for language editing and refinement of the manuscript text. The tool will be applied only for proofreading and improving clarity of language; all scientific content, analyses and interpretations will be produced by the authors.

## Summary of design rationale

This study is designed to test preregistered hypotheses that there are sex differences in stroke characteristics, management and outcomes among AF patients using a confirmatory Registered Report framework. The use of a large, routinely collected registry data allows for the systematic evaluation of these questions while minimizing selection bias and enhancing generalizability. By focusing the primary confirmatory analyses on patients with AF presenting with LVO stroke treated with EVT, the study targets a clinically homogeneous population with well-defined treatment pathways. This design reduces clinical heterogeneity and strengthens interpretability of observed differences. To assess broader relevance beyond the EVT population, exploratory analyses will additionally include all patients with AF and ischaemic stroke recorded in the Norwegian Stroke Registry. Secondary exploratory aims will examine age, comorbidity burden, treatment delays and health system factors as potential contributors to observed sex differences.

Together, this design enables transparent and rigorous assessment of whether sex differences are present in stroke care and outcomes, while ensuring that both confirmatory and exploratory findings are interpreted within a clearly prespecified analytic framework.

### Design considerations

A structured overview of hypotheses, outcomes, covariates and planned statistical models is provided in Table 1 (Analysis Overview). Additional details on theoretical rationale, sensitivity considerations and interpretation of potential outcomes are provided in the Supplementary material.

**Table 1:** Analysis overview

Hypothesis	Analysis type	Population	Outcome(s)	Predictors/covariates	Statistical model	Planned adjustment	Statistical inference
H1: Sex differences in stroke characteristics, treatment and outcomes	Con	AF+LVO stroke, EVT treated	Stroke severity (NIHSS), functional outcome (mRS at 3 mo), mortality (in-hospital, 3 mo, 1 yr, 2 yr and 3 yr)	Sex	Chi-square, *t*-tests, logistic regression, Cox (mortality)	Age, comorbidities, stroke severity, treatment	Two-sided family wise α = 0.05 (Holm–Bonferroni); adjusted OR, HR, 95% CI
H2: Women are older and have more comorbidities	Exp	All AF stroke	Age, comorbidity burden	Sex	*t*-tests, chi-square	—	Descriptive + significance testing
H3: Sex differences in pre-hospital and in-hospital delays and reperfusion therapy	Exp	AF+LVO stroke	Time to treatment (onset-to-door, door-to-imaging, door-to-needle, door-to-groin), IVT/EVT	Sex	*t*-tests, logistic regression	Age, comorbidities	α = 0.05
H4: Women are less likely to be adequately anticoagulated	Exp	AF+LVO stroke	Adequate anticoagulation at onset	Sex	Chi-square, logistic regression	Age, comorbidities	α = 0.05
H5: Mediators of sex differences in outcome	Exp	AF+LVO stroke	Functional outcome, mortality	Age, comorbidity and system factors	Mediation analysis, interaction terms	Age, comorbidities	Bootstrap indirect effects, α = 0.05

Overview of preregistered confirmatory (Con) and exploratory (Exp) hypotheses, corresponding outcomes, predictors and planned statistical models. This table summarizes the main analytic strategy for Stage 1 of the Registered Report.

## Limitations

This is a retrospective, observational study, which limits causal inference. While the Norwegian Stroke Registry offers broad coverage, variation in data completeness and accuracy across hospitals may influence results. Classification of adequate anticoagulation depends on available documentation and may be affected by misclassification, and some patients may have clinical contraindications to anticoagulation that are not captured in registry data. Residual confounding from unmeasured variables may persist despite multivariable and mediation analyses. Follow-up for 1-, 2- and 3-year mortality will depend on successful linkage to the Norwegian Cause of Death Registry and/or availability of data in hospital records.

## Supplementary Material

fcag386_Supplementary_Data

## Data Availability

The dataset from Stavanger University Hospital is complete and available to the research team. The descriptive analyses have been conducted to assess data completeness and feasibility; however, no confirmatory or hypothesis-driven analyses related to sex differences or anticoagulation status have been performed. National-level data from the Norwegian Stroke Registry exist and will be used to extend and assess the generalizability of findings from the local cohort. These data have not yet been accessed by any member of the research team. The access will be sought following Stage 1 in-principle acceptance, and all analyses involving national data will be conducted thereafter in full accordance with the preregistered analysis plan.

## Data and code availability

The study uses routinely collected clinical and registry data from Stavanger University Hospital and the Norwegian Stroke Registry. Due to Norwegian data protection legislation and registry governance, de-identified individual-level data cannot be made publicly available but may be accessible upon request, subject to approvals from the relevant data controllers and ethics authorities. The full statistical analysis code and prespecified scripts will be made available via the Open Science Framework repository associated with this Registered Report at the time of Stage 2 publication (URL/DOI to be added upon acceptance).

## Preregistration

The complete protocol and statistical analysis plan for the confirmatory analyses will be preregistered on the Open Science Framework (OSF) prior to accessing national registry data. A DOI or registration link will be added upon acceptance.

Reporting guideline: at Stage 2, we will provide the appropriate reporting guideline checklist(s) for an observational cohort study using routinely collected health data (STROBE and, where applicable, the RECORD extension).
