# Supplementary material for: Stage 1 Registered Report: sex differences in anticoagulation and stroke outcomes among patients with atrial fibrillation: a registry-based cohort study
Source: Brain Commun. 2026 Oct 22;8(6):fcag386. doi: 10.1093/braincomms/fcag386 (PMC13722935; doi:10.1093/braincomms/fcag386)
Supplement: Supplementary file 1 [file fcag386_supplementary_data.pdf]

Supplementary Material

Manuscript title: *Stage 1 Registered Report: Sex Differences in Anticoagulation and Stroke Outcomes Among Patients with Atrial Fibrillation: A Registry-Based Cohort Study*

1. Additional Methodological Details

This Supplementary Material provides additional details on variable definitions, planned sensitivity analyses, and subgroup analyses referred to in the main manuscript.

1.1 Variable definitions and data sources

Variable	Definition	Source
**Sex**	Biological sex at birth	All datasets
**Adequate anticoagulation**	Therapeutic INR ≥ 2.0 for VKA users, or documented DOAC use within 48 h before stroke onset	Local registry / Norwegian Stroke Registry
**NIHSS**	Stroke severity at admission	Registry / Hospital records
**mRS**	Modified Rankin Scale at 3 months	Registry
**Treatment times**	Onset-to-door, door-to-imaging, door-to-needle, door-to-groin puncture	Hospital records
**Mortality**	In-hospital, 3-month, 1-year, and 2-year mortality	Hospital records / Cause of Death Registry
**Health system factors**	Weekday/weekend admission, inter-hospital transfer, year of treatment	Registry / Hospital data

2. Sensitivity and Subgroup Analyses

- **Anticoagulation:** Results will be checked using alternative definitions of adequacy (e.g. DOAC use within 24 h).
- **Functional outcome:** The primary outcome (mRS 0–2) will also be analysed using ordinal logistic regression as a sensitivity check.
- **Missing data:** Multiple imputation will be compared with complete-case analysis.
- **Subgroups:** Analyses will be stratified by
    - Age (<75 vs ≥75 years)

Følsomhet Intern (gul)

    - Stroke severity (NIHSS <15 vs ≥15)
- **Equivalence testing:** Equivalence testing will be conducted in the event of non-significant results to assess practical similarity between groups.

3. Mediation and Interaction Analyses

Bootstrapped mediation models will test whether age, comorbidities, or healthcare delivery factors mediate the relationship between sex and outcomes.
Interaction terms (sex × age, sex × comorbidity) will explore whether the strength of associations differs between subgroups.

4. Interpretation Framework

If sex differences in anticoagulation, treatment, or outcomes are observed, they will be interpreted in light of clinical practice patterns and potential biological explanations.
If no significant differences are found, results will be interpreted as evidence of treatment equity in the studied population.

Følsomhet Intern (gul)
